# Colossal Parotid Tumors: A Diagnostic and Surgical Challenge

**DOI:** 10.7759/cureus.14539

**Published:** 2021-04-18

**Authors:** Ankit Jain, Mahalingam Sudharshan, Chellappa Vijayakumar, Uday Kumbhar, Vishnu Prasad Nelamangala Ramakrishnaiah

**Affiliations:** 1 Surgery, Jawaharlal Institute of Postgraduate Medical Education and Research (JIPMER), Puducherry, IND

**Keywords:** giant salivary gland tumors, warthin's tumor, pleomorphic adenoma, parotid, superficial parotidectomy, facial nerve palsy

## Abstract

Tumors of the salivary glands constitute 3% of all head and neck tumors. The parotid gland (PG) is the most common site involved in 85% of cases. PG tumors' size varies from a few millimeters to several centimeters and is about 2-6 cm on average. However, because of insidious growth and asymptomatic nature, untreated tumors of the PG can attain large size. Pleomorphic adenoma (PA), as large as 33 cm in size or 26.5 kg in weight, has been reported in the literature. Similarly, untreated Warthin’s tumor (WT) rarely becomes giant, size up to 20 cm is reported. Giant PG tumors are commonly symptomatic and have a rare tendency to become malignant.

We are reporting two giant PG tumors with different histopathological diagnoses, PA and WT of size 15x15 cm and 10x8 cm, respectively. Therefore, with a size of 10 cm, our case is the second-largest WT reported in the literature. Both the giant PG tumors were present for 15-20 years, and mild pain and discomfort were the only symptoms. We had the differential preoperative tissue diagnosis in fine-needle aspiration cytology (FNAC) because of varying consistency. Contrast-enhanced computed tomography (CECT) and magnetic resonance imaging (MRI) of the neck were done for these cases for preoperative planning. Compared to the former, the latter was more informative about nerve involvement preoperatively. Both the patients underwent superficial parotidectomy, and meticulous dissection was done to identify and safeguard the facial nerve and its branches. We had a challenge in closing the flaps, which was achieved with an acceptable cosmetic outcome. Both the patients were discharged in stable condition with minimal facial nerve weakness.

## Introduction

Pleomorphic adenoma (PA) is the most common salivary gland tumor (70%), followed by Warthin’s tumor (WT) (6%-10%) [1]. The parotid gland (PG) is the most common site of salivary gland tumors [2]. The size of PG tumors varies from few millimeters to several centimeters and is about 2-6 cm on average [3-5]. However, because of insidious growth and asymptomatic nature, untreated tumors of the PG can attain a large size. Giant parotid gland tumors (GtPGTs) weighing as much as 26.5 kg are reported in the literature [6]. Herein, we report two GtPGTs with a different histopathological diagnosis, PA and WT.

## Case presentation

Case summary 1

A 60-year-old male presented with complaints of swelling over the right side of the face for 20 years. The swelling remained static initially, which gradually increased in size for the last five years and was not associated with any sudden increase in size. He had no pain complaint, difficulty in mouth opening, facial weakness, facial paraesthesia, halitosis, altered salivary flow, or discharge from the swelling during its progression.

**Figure 1:**
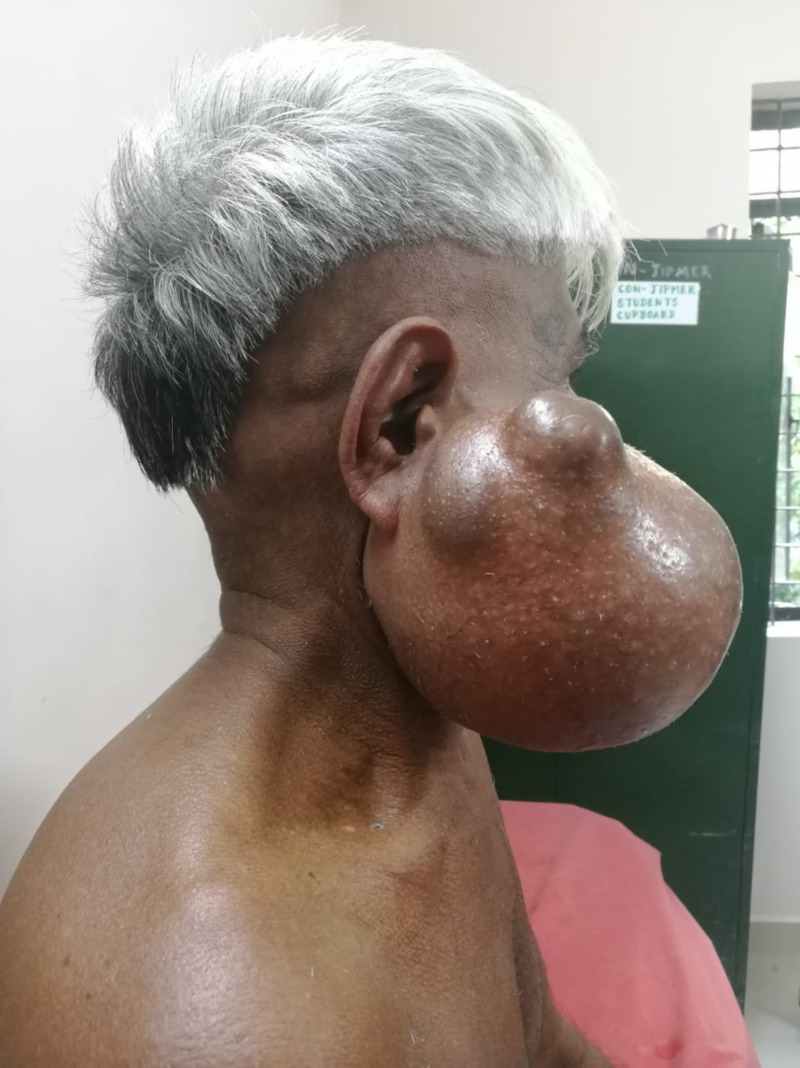
Clinical image of the patient (Case 1) showing a large right-sided parotid swelling

On local examination, an ovoid mobile swelling of size 15x15 cm was noted below, behind, and in front of the right ear with obliteration of the retro-mandibular furrow and lifting of the right ear lobule with no local warmth or tenderness. The swelling had well-defined margins, a smooth surface, a variegated consistency predominantly fluctuant cystic area, and a firm solid area in its posterosuperior part. There were two soft outpouchings noted on the surface of swelling, which were compressible. The swelling was not fixed to the underlying muscles or the overlying skin except in the area of outpouching nodules. The skin over the swelling had normal facial hairs and had no dilated veins, scars, sinuses, or fistulae (Figure 1).

**Figure 2:**
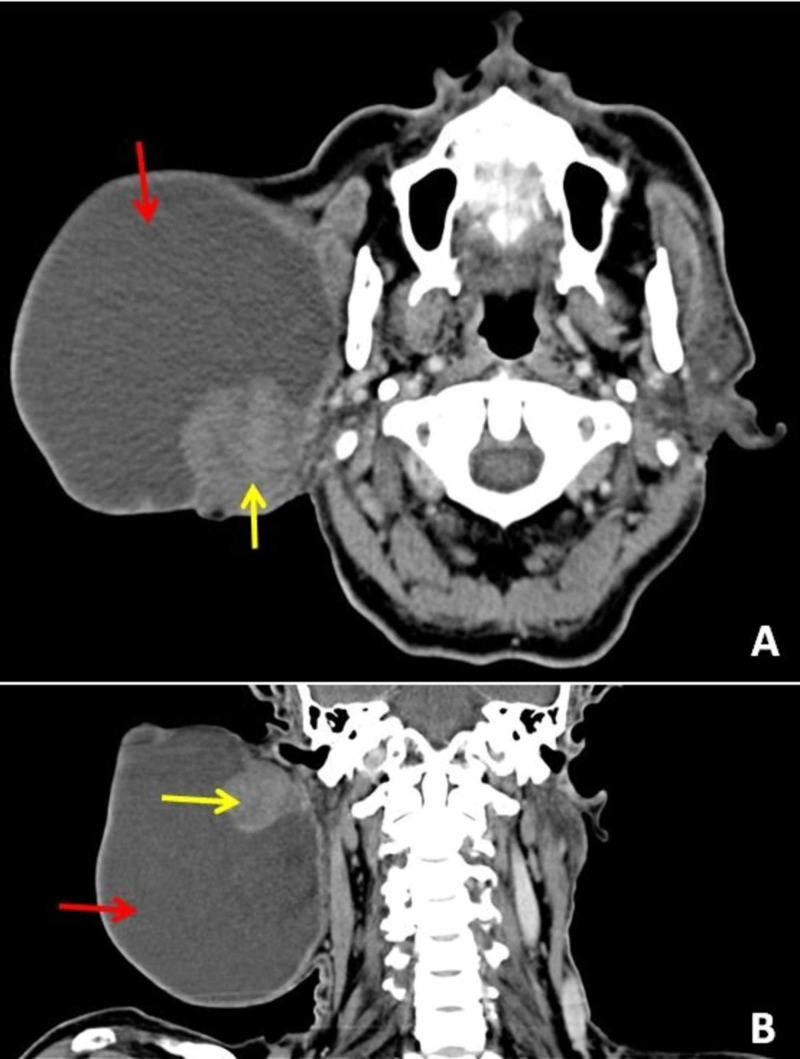
Post-contrast CECT A: Coronal, and B: Axial view showing a well-defined fluid attenuating cystic lesion (red arrow) with an eccentric, enhancing nodular solid component (yellow arrow) in the right parotid region. CECT: contrast-enhanced computed tomography

His intraoral examination revealed a normal deep lobe, parotid duct, and its opening. There was no apparent facial asymmetry or weakness of facial muscles. Contrast-enhanced computed tomography (CECT) revealed a well-defined, hyperdense, fluid-attenuating cystic lesion of size 11x13x12 cm with an eccentric enhancing nodular solid component of size 4x5 cm in the right parotid region. The deep lobe of the parotid was normal. There was no involvement of the deeper structures and vessels of the neck. There was no significant lymphadenopathy (Figure 2).

**Figure 3:**
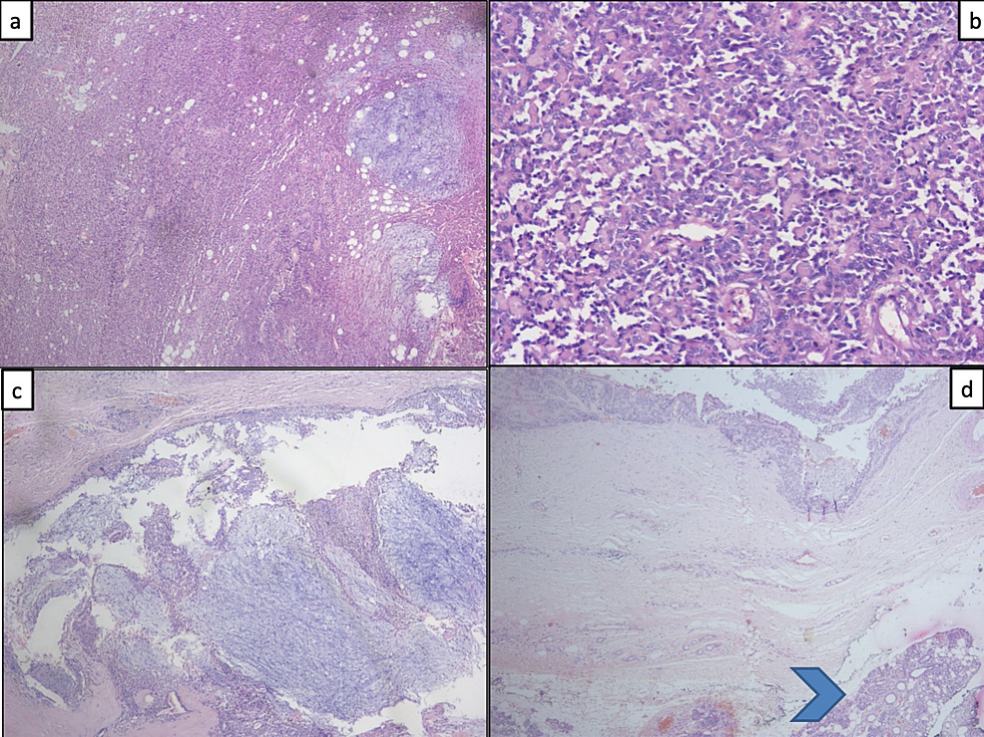
Photomicrographs of histopathological sections a) Tumor composed of solid sheets of epithelial cells and focal chondromyxoid background (H&E X 40); b) High-power sections of sold areas show nests and cords of epithelial cells admixed with few myoepithelial cells (H&E X 200); c) Focal areas show a cyst composed of predominantly chondromyxoid areas admixed with a few cords of epithelial cells (H&E X 100); d) Show a tumor with normal seromucinous glands of the parotid (blue arrowhead) (H&E X 100) H&E: Hemotoxylin and Eosin

Fine-needle aspiration cytology (FNAC) from the lesion showed features suggestive of PA. The patient underwent a right superficial parotidectomy. Blair’s incision was given with a 5 cm extension in the cervical skin crease because of significant swelling. Intraoperatively, the facial nerve's zygomatic branch was found to be traversing through the cyst and was sacrificed. The rest of the branches were identified and preserved. There was an excess of thinned and redundant skin, which was trimmed to achieve cosmetically acceptable closure. Postoperative microscopic examination revealed PA without any evidence of malignancy (Figure 3).

Case summary 2

A 63-year-old male presented with complaints of swelling in the right side of the neck extending to the right cheek for 15 years. There was no sudden increase in the size of the swelling. He had no other complaints similar to Case 1.

On local examination, an irregular, non-tender, mobile swelling of size 10x8 cm was noted below, behind, and in front of the right ear with obliteration of the retro-mandibular furrow and lifting of the right ear lobule. The swelling had well-defined margins, a smooth surface, and variable consistency with cystic and solid areas admixed. It was not fixed to the masseter, sternocleidomastoid, or overlying skin. The skin over the swelling and his intraoral examination were clinically unremarkable. There was no evidence of facial palsy or lymph nodes in the neck.

**Figure 4:**
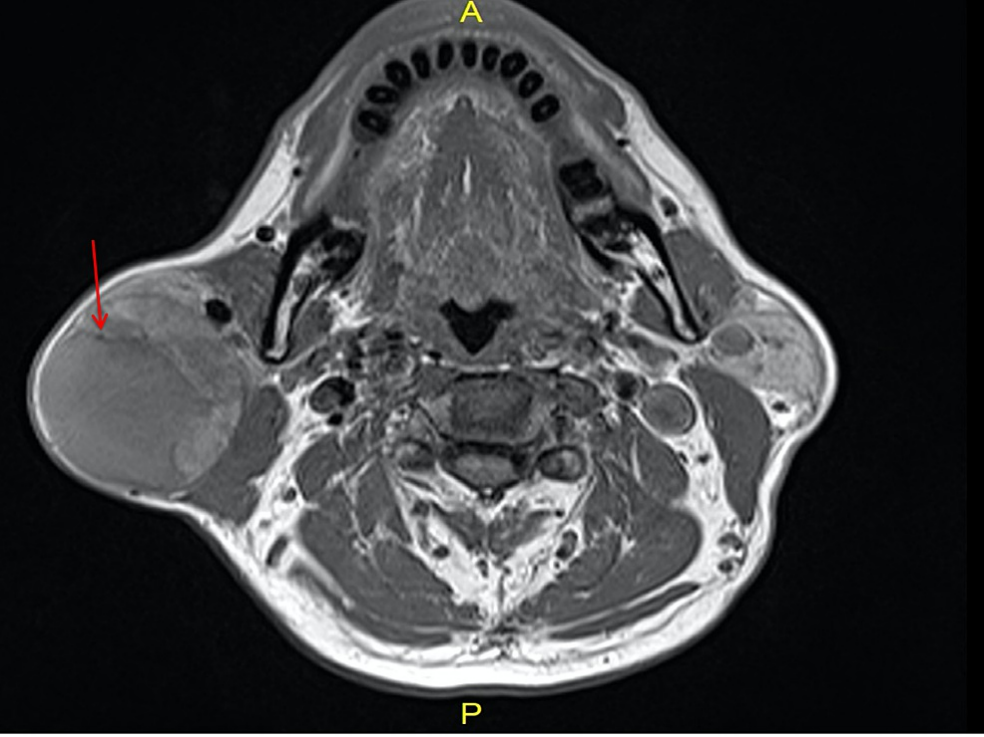
T1 axial image, T1 intermediate to mildly hyperintense cystic component of lesions (red arrow) - possibly of mucoid content

Magnetic resonance imaging (MRI) of the neck showed a well-circumscribed, solid-cystic lesion within the posteroinferior part of the right parotid gland's superficial lobe with no extension to the deep lobe measuring 8.5x6.4x8 cm. Peripheral solid areas of the lesion along the superior and medial aspects show diffusion restriction with post-contrast enhancement. There was a presence of T2 intermediate to hyperintense signal with scattered T1/T2 hyperintense cysts. A T2 hypointense T1 intermediate to mildly hyperintense cystic component of the lesion (measures 4.0x3.8 cm^2^) with possibilities of mucoid content was also noted. The lesion was abutting the retromandibular vein and displacing it anteriorly. There were no suspicious deep cervical lymph nodes noted (Figure 4).

**Figure 5:**
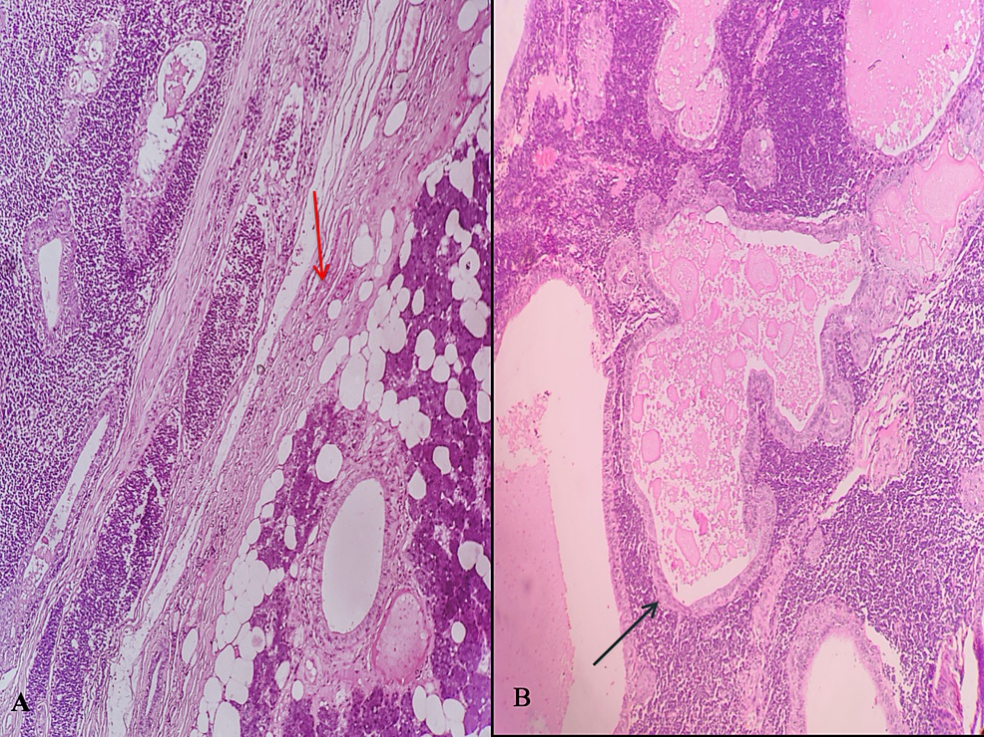
Photomicrographs of histopathological sections A: Interface between the salivary gland parenchyma and the tumor - red arrow (H&E 40); B: Cystic structures lined by benign bilayered epithelium surrounded by a dense lymphoid stroma - black arrow (H&E X 100) H&E: Hematoxylin and Eosin

FNAC showed differentials of WT and lymphoepithelial cyst. He underwent right superficial parotidectomy with standard Blair’s incision. Meticulous dissection was done to identify and safeguard the facial nerve and its branches. During his immediate postoperative period, he developed facial nerve neuropraxia, which was resolving well with physiotherapy. The histopathology of the specimen revealed features of WT (Figure 5).

## Discussion

The common site of the tumor in PG is the superficial lobe or tail of the gland; however, deep lobe involvement is also seen. The size of PG tumors varies from a few millimeters to several centimeters and is about 2-6 cm on average [3-5]. The size of reported cases of giant WT was found to be smaller than PA. WT up to 8 cm is reported in the literature, with only one tumor of 20 cm size [1]. Therefore, with a size of 10 cm, our case is the second-largest WT reported in the literature. This size difference could be because of WT's peak incidence in the 6th-7th decade, with an incidence as low as 6% below 40 years of age [1,7]. The dimensions of these GtPGTs can range from 15-20 cm, with 33 cm as the maximum size reported in the literature [8-10]. These tumors can weigh in kilograms. As per a series of 31 cases published by Schultz-Coulon, the weight of such tumors varied from 1-26.5 kg [6].

The tumors of PG are generally slow-growing with no symptoms other than aesthetic implications. Therefore, these tumors are generally ignored by patients in the initial stages. Various factors are responsible for this neglect [2,6-8]. Therefore, continuous non-treatment for decades altogether leads to these tumors growing to a disproportionately large size. In our case, both patients belonged to a remote village and didn’t have access to a proper health care facility. Patients generally tolerate this slow-growing mass for years and present for treatment when it becomes symptomatic. Therefore, patients tend to be in their fifth or sixth decade [7]. However, cases are reported as young as the 25-year-old patient [6]. A very large swelling can assume the appearance of a “double head,” which can have psycho-social issues, especially in rural areas [8]. However, fascial nerve weakness or paralysis is rarely seen even with such a large tumor size.

Ultrasonography (USG) is the initial investigation of choice. But USG has certain limitations such as difficulty studying the deep parotid lobe because of the mandible acoustic shadow, inability to visualize the facial nerve, retropharyngeal and deep neck lymphadenopathies, and the difficult to identify the involvement of surrounding tissue [4,11]. This problem is compounded in GtPGTs due to their size. Therefore, MRI is the preferred investigation for such GtPGTs, primarily due to its ability to provide excellent morphological and volume assessment and a precise definition of the relationship with adjacent structures [11]. This information becomes more critical in cases of GtPGTs because distortion of the surrounding anatomy is expected due to their large size [1]. On MRI, GtPGTs tend to be well-capsulated, lobulated, and non-homogenous in appearance due to areas of necrosis, hemorrhagic, and cystic degeneration with or without calcification [3,11]. There is also increased vascularity of glands with multiple feeder vessels, commonly arising from the external carotid artery or its branches, seen on imaging [2,7-8,10]. CECT can also be used only in cases where MRI is contraindicated or not available [11].

FNAC is a rapid and useful tool for cytological diagnosis. The risk of dissemination of neoplastic cells along the needle tract and facial nerve injury is also negligible [11]. However, the large size of these GtPGTs increases the risk of having a false negative or inconclusive report owing to the inability to sample the representative areas. The majority of PA cases are probably because it is the most common PG tumor [1]. However, cases of WT, malignant transformation in PA, malignant PG tumor, or secondary carcinoma in PA are also reported [4,6,9-10].

Although benign, tumors of the salivary gland, such as PA and WT, mandates excision because of the risk of malignant transformation. The risk of malignant transformation in PA increases from 1.6% in tumors with less than five years duration, to 9.5% for those presenting for more than 15 years [3,5]. The same risk in WT is 0.3% [1]. The risk of transformation increases with recurrence after surgery. Therefore, excision with uninvolved margins is necessary. Since GtPGTs are both long-standing as well as symptomatic at presentation, excision is mandatory even if metastatic malignancy is suspected [10]. However, there are specific problems inherent to the surgical management of these tumors. First, tumors can be approached with modified Blair’s incision, but good cosmetic outcomes mandate properly planned skin incision with the excision of redundant or fixed or involved skin [1,7]. Second, large feeder vessels predispose to an increased risk of intraoperative and postoperative hemorrhage [7-8,10]. Many authors have suggested the role of preoperative embolization, but owing to the rarity of GtPGTs, no conclusive data are available [7-8,10]. Third, such large tumors distort the anatomy of surrounding structures, including splaying of facial nerve branches, exposing them to injury. However, these tumors are seen to separate easily from surrounding tissue [2,7,10]. Therefore, with meticulous dissection, the fascial nerve injury can be kept to a minimum.

## Conclusions

PG tumors usually remain untreated and grow to an enormous size due to various factors such as poor socioeconomic conditions, lack of access to healthcare, and fear of surgery. GtPGTs can be benign as well as malignant. Also, long-standing GtPGTs become symptomatic and have more risk of malignancy, thus mandating surgical excision. Therefore, excision with uninvolved margins should be considered for all GtPGTs. Surgery needs proper preoperative planning to minimize intra and postoperative complications.
